# Hollow silica reinforced magnesium nanocomposites with enhanced mechanical and biological properties with computational modeling analysis for mandibular reconstruction

**DOI:** 10.1038/s41368-020-00098-x

**Published:** 2020-11-17

**Authors:** Somasundaram Prasadh, Vyasaraj Manakari, Gururaj Parande, Raymond Chung Wen Wong, Manoj Gupta

**Affiliations:** 1 Faculty of Dentistry, National University of Singapore, 9 Lower Kent Ridge Road, Singapore, Singapore; 2 Department of Mechanical Engineering, National University of Singapore, 9 Engineering Drive 1, Singapore, Singapore

**Keywords:** Biomedical engineering, Biomedical materials, Implants

## Abstract

The present study investigates Mg-SiO_2_ nanocomposites as biodegradable implants for orthopedic and maxillofacial applications. The effect of presence and progressive addition of hollow silica nanoparticles (0.5, 1, and 1.5) vol.% on the microstructural, mechanical, degradation, and biocompatibility response of pure Mg were investigated. Results suggest that the increased addition of hollow silica nanoparticles resulted in a progressive increase in yield strength and ultimate compressive strength with Mg-1.5 vol.% SiO_2_ exhibiting superior enhancement. The response of Mg-SiO_2_ nanocomposites under the influence of Hanks’ balanced salt solution revealed that the synthesized composites revealed lower corrosion rates, indicating rapid dynamic passivation when compared with pure Mg. Furthermore, cell adhesion and proliferation of osteoblast cells were noticeably higher than pure Mg with the addition of 1 vol.% SiO_2_ nanoparticle. The biocompatibility and the in vitro biodegradation of the Mg-SiO_2_ nanocomposites were influenced by the SiO_2_ content in pure Mg with Mg-0.5 vol.% SiO_2_ nanocomposite exhibiting the best corrosion resistance and biocompatibility when compared with other nanocomposites. Enhancement in mechanical, corrosion, and biocompatibility characteristics of Mg-SiO_2_ nanocomposites developed in this study are also compared with properties of other metallic biomaterials used in alloplastic mandibular reconstruction in a computational model.

## Introduction

Bioresorbable metals are a class of path-breaking biomaterials that have reshaped the nature of metallic biomaterials from bioinert regime to bioactive regime and multi-bio functional (anti-bacterial, anti-proliferation, and anti-cancer) regime^1^. Encouraged by this development, in recent years, magnesium (Mg)-based alloys have been extensively researched for orthopedic and maxillofacial osteosynthesis^1–9^. Magnesium has the advantage of being biocompatible and bioresorbable within the human body when compared with currently used permanent osteosynthesis systems manufactured from titanium alloys^1^. Using a biodegradable metal-like magnesium avoids further surgical intervention to remove the implants after desirable bone regeneration, thereby reducing the associated expenses and risk of further surgical complications. The key feature of magnesium compared with its counterpart metals is that magnesium has a similar elastic modulus (40–45 GPa) to the cortical bone (~15–30 GPa) and this reduces the stress shielding effect owing to the mismatch in elastic modulus between the cortical bone and the implant^10–12^. In addition, magnesium is osteoconductive and thereby facilitates bone cell growth and has also been shown to contribute to cell attachment^13^. Guo et al.^14^ implanted MC (mineralized collagen) and Mg-Ca-MC on the buccal plates of a patient’s mandible and post-implantation bone formation was evaluated for 12 and 24 weeks. After 24 weeks, patients who had been given the Mg-Ca-MC implant exhibited more dense and compact cortical bone formation when compared with the MC implant. Mg accelerated the growth of new bone and repaired the alveolar ridge of the buccal bone defect. In another study to evaluate the osteogenic potential of Mg, Wang et al.^14,15^ extracted the second and third premolar of the mandibular canines and implanted Mg-Sr on the buccal fenestration bone defect. Increased bone formation and bone mineral density were observed in patients with Mg-Sr implants compared with patients with MC implants.

However, usage of magnesium in the clinical application has been limited by its low strength, poor formability, lower fatigue resistance, and rapid degradation in high chloride physiological environment^16,17^. The addition of nano-length scale reinforcements (<3 vol.%) into the Mg matrix has been shown to overcome these limitations with simultaneous improvements in strength, ductility, and corrosion resistance of the material^18–22^. For example, Kujur et al.^12^ synthesized Mg-CeO_2_ nanocomposites with different vol.% (0.5, 1, and 1.5 vol.%) of cerium oxide nanoparticles (NPs) and reported that Mg-CeO_2_ nanocomposites exhibited better mechanical properties than commercially available Mg alloys such as WE43, AZ31, ZK21, and AZ91. In addition to CeO_2_, various other metal oxide NPs like Sm_2_O_3_, ZnO, ZrO_2_, Al_2_O_3_, and TiO_2_ have also been reinforced with Mg to achieve superior performance owing to their high chemical stability, a high degree of biocompatibility and non-toxicity^18,19^. Ong et al.^23^ studied the effect of one such metal oxide NPs on the cytotoxicity response of pure Mg. It was observed that addition of 2.5 vol.% TiO_2_ NPs had little to no effect on the cytotoxicity behavior of the Mg matrix, indicating their suitability for implant applications.

Silica (SiO_2_) has been utilized in various applications ranging from microelectronics to food and pharmaceutical industries^24^. Further, owing to their high degree of biocompatibility, SiO_2_ NPs have found applications in biosensors, drug delivery systems, and enzyme immobilization^25–27^. Also, being the major constituent of bioglass (~45 wt.%), it has been extensively investigated for biomedical applications^28^. In a recent study of Wan et al.^29^, incorporation of 45S5 bioglass (30–75 µm) to pure Mg resulted in a significant enhancement in the compressive strength to 100 MPa (~18% greater than pure Mg) with a minor reduction in ductility values. Further, Beck et al.^30^ evaluated the bone mineral density (BMD) in mice by bioactive silica NPs (SiO_2_ NPs). From a biocompatibility perspective, SiO_2_ NPs (silica NPs) were observed to have positive stimulatory effects on osteoblasts in vitro and increased bone density in vivo in mice. It was observed in the study that >95% of silicon was passed out of the body through feces and urine indicating a rapid and near full clearance of NPs by the animal^30^. Their results also showed the addition of SiO_2_ NPs stimulated osteoblastic differentiation, decreased osteoclastic activity, and increased BMD in mice. Also, SiO_2_ NPs reinforced chitosan microparticles for bone regeneration showed excellent biocompatibility, increased cell proliferation, and increased osteogenic gene expression^31^.

Hollow SiO_2_ NPs have a high-specific surface area, low density, good biocompatibility, and low toxicity^32^. Hollow SiO_2_ NPs are extensively researched in smart biological applications such as enzyme supporters^25^ and biosensors^26^ and for controlled drug release and delivery^15,27^. In a recent study, Yu et al.^24^ observed that hollow SiO_2_ NPs exhibited no signs of toxicity and could be safely metabolized and tolerated in mice without longstanding cytotoxicity. Their work provided a unique perspective on the application of hollow SiO_2_ NPs for cancer therapy to further broaden the horizon of nanomaterials used for biomedicine. Hence, hollow SiO_2_ NPs (10–20 nm) currently chosen for this study can be considered a safe and valid reinforcement for Mg targeting biomedical implant applications. A literature survey reveals no previous attempt made so far to study the effects of hollow silica (SiO_2_) NPs on the microstructural, mechanical, degradation, and biocompatibility properties of pure Mg. Therefore, Mg-(0.5, 1.0, and 1.5 vol.%) hollow SiO_2_ nanocomposites were synthesized using disintegrated melt disposition (DMD) method. The effects of reinforcement on the cell proliferation, cytotoxicity, mechanical strength, and corrosion behavior are presented.

The outcomes of this study are initial results to validate the applicability of Mg nanocomposites for potential applications in orthopedic and craniomaxillofacial osteosynthesis systems. Finally, we expect that our results will help in ultimately extending the scope of Mg-based bioresorbable materials into clinical translation for osteosynthesis and alloplastic bone replacement that can be resorbed and replaced eventually with bone.

## Results

### Characterization of microstructure and mechanical testing

The microstructure characterization is illustrated in Fig. S1 and Fig. S2. The results of grain size measurements are shown in Table S1. Nanocomposites predominantly showed the equiaxed nature of grains with significant grain refinement compared with pure Mg. Mg-1.5 SiO_2_ exhibited an average grain size of 16 μm, which is ~ 55.6% finer compared with pure Mg. Further, uniform dispersion of the NPs was observed in the Mg matrix with good interfacial integrity and minimal agglomeration for all the compositions (Fig. S2).

**Fig. 1:**
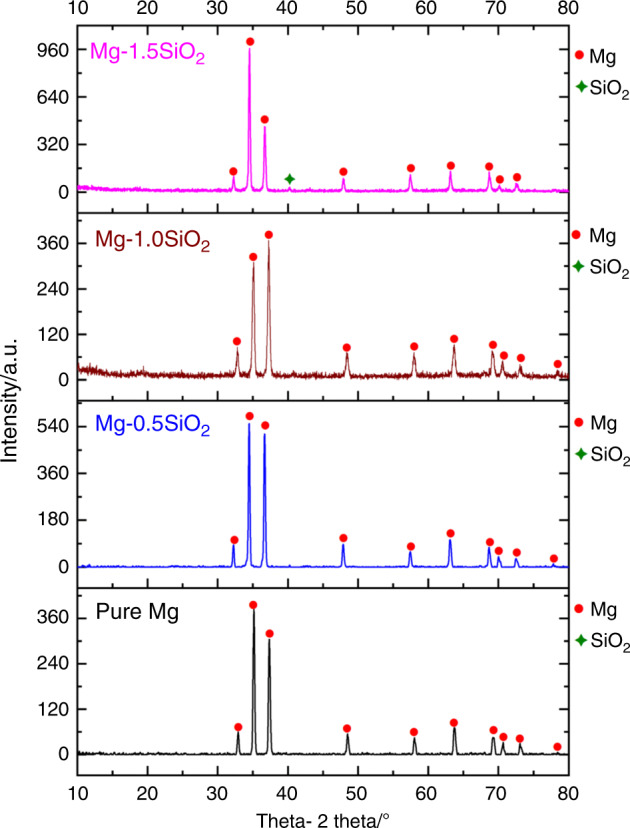
X-ray diffractograms of pure Mg and Mg-SiO_2_ nanocomposites taken along the longitudinal direction of hot extruded samples. X, Y, Z represent 2θ = 32°, 34°, and 36° corresponding to (10–10) prism, (0002) basal, and (10–11) pyramidal planes, respectively

X-ray diffractogram obtained on performing X-ray diffraction studies are shown in Fig. 1. The prominently visible peaks are predominantly of Mg. SiO_2_ peaks for lower volume percent additions (0.5 and 1.0 vol.%) were not distinctly visible. This is attributed to the X-ray machine’s limitation to accurately distinguish reinforcements with a low-volume fraction (<1%). However, the distinct peaks of SiO_2_ were observed with 1.5 vol.% addition as seen in Fig. 1. In addition, the presence of SiO_2_ NPs can be further confirmed from the high-resolution microstructural characterization (Fig. S2). The absence of additional peaks in Mg-SiO_2_ nanocomposites suggests that no interfacial reaction/secondary phase formation occurred between Mg and SiO_2_ NPs during DMD processing and hot extrusion. From the longitudinal X-ray diffractograms (Fig. 1) (Table S2), the characteristic peaks for the as-extruded pure Mg and Mg-SiO_2_ nanocomposites, the characteristic peaks observed of hexagonal close-packed Mg crystal at angle 2θ = 32° (prismatic), 34° (basal), and 36° (pyramidal) planes. Although pure Mg displayed a strong basal texture, for Mg-(0.5, 1 vol.%) SiO_2_ nanocomposites, the intensity corresponding to the pyramidal plane was observed to increase compared with that of the basal plane indicating a higher level of randomization in the texture.

The compressive properties and their stress–strain relationship for the developed Mg-SiO_2_ nanocomposites are shown in Table S3 and Fig. S3, respectively. The incorporation of hollow SiO_2_ NPs into the magnesium matrix leads to a progressive improvement in the 0.2% yield strength (CYS) and ultimate compressive strength (UCS) values in all compositions. Mg-1.5 vol.% SiO_2_ nanocomposite exhibiting the maximum CYS and UCS values of ~128 MPa and ~378 MPa, respectively, the highest among all nanocomposites.

Simultaneous improvements in the fracture strain values were also observed in the case of Mg-0.5 SiO_2_ and Mg-1.0 SiO_2_ nanocomposites (max: ~23.8%) when compared with that of pure Mg (~21.2%). However, with the further addition of SiO_2_ NPs (1.5 vol.%), a reduction in fracture strain value (~18.1%) was observed. From Table 1, the compressive properties of the developed nanocomposites matches/exceeds previously reported commercial and recently researched Mg alloys. Further, the mechanical properties of the developed nanocomposites closely match with regards to bone material, suggesting their potential as osteosynthesis implants used in craniomaxillofacial surgery.

### Immersion studies

**Fig. 2:**
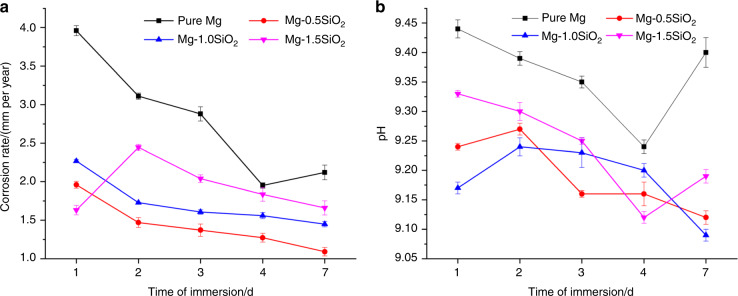
The immersion response of the synthesized samples. **a** Calculated corrosion rates based on weight loss and **b** pH measurements from immersion testing

**Fig. 3:**
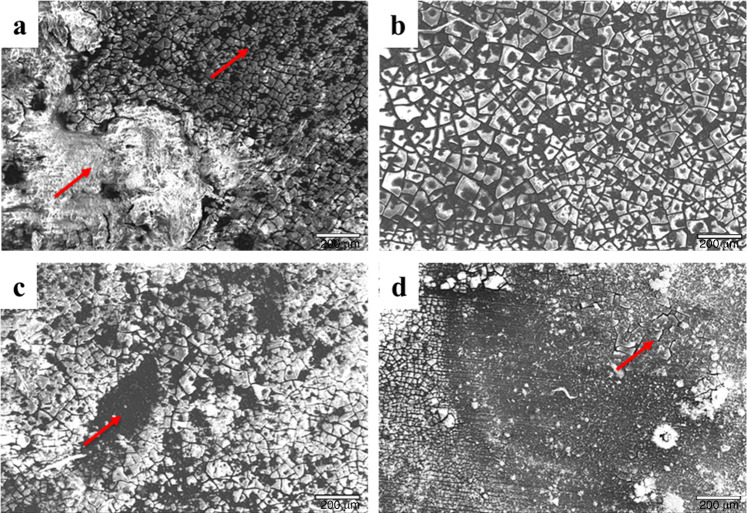
Scanning electron microscope images of **a** pure Mg, **b** Mg-0.5 SiO_2_, **c** Mg-1.0 SiO_2_, and **d** Mg-1.5 SiO_2_ nanocomposites after 7 days of immersion (red arrows indicating the corroded and non-corroded areas). Magnification, ×75. Scale bars are 200 μm

The immersion response of the synthesized samples and SEM of the corroded surfaces are shown in Fig. 2 and Fig. 3. The energy dispersive spectroscopy (EDS) mapping of the corroded surface is shown in Fig. S4. The corrosion rates and pH values were measured at every cycle. The pH values for all the samples increased drastically at the end of 24 h with the pH values being in the range of ~9.2–9.4. The high initial pH increase can be attributed to the interaction of Mg and Mg-based materials with physiological environments wherein high interaction is observed for the initial 12–24 h^30^.

This interaction leads to the anodic dissolution of Mg and Mg-based nanocomposites releasing Mg^2+^ ions into the solution. The higher the rate of dissolution, the greater is the amount of Mg^2+^ released and higher pH values are observed. However, pH values from day 2 to 7 for all the samples reveal steady pH values, and no substantial increase or decrease in the values was observed. The absolute pH values of the nanocomposites were observed to be lesser than that of pure Mg. This suggests that the uniform presence of the NP has resulted in enhanced dynamic passivation of the composite samples from day 2 to 7 thereby keeping the pH increase/decrease in control. The corrosion rates were calculated as per Eq. (1), where, time conversion coefficient, *K* = 8.76 × 10^4^, *W* is the change in weight pre and post immersion (g), *A* is the surface area of the cylinder exposed to the immersive medium (cm^2^), *T* is the time of immersion (h), and *D* is the experimental density of the material (g·cm^−3^)^16^.

1 $$Corrosion\,rate = \frac{{K \times W}}{{(A \times T \times D)}}$$

The corrosion rates at the end of day 1 for pure Mg was ~3.9 mm/y. In comparison, the nanocomposites displayed lower corrosion rates with Mg-1.5 vol.% SiO_2_ nanocomposites showing the least value of ~1.6 mm/y. The corrosion rates of the samples decreased progressively for all the samples except for Mg-1.5 vol.% SiO_2_ nanocomposite, which shows a slight initial increase. Mg-0.5 vol.% SiO_2_ nanocomposite displayed the best response with near-uniform corrosion rate from day 2 to day 7. Mg-1 vol.% and Mg-1.5 vol.% SiO_2_ nanocomposites also displayed a decreased corrosion rate, however, the trend observed was not progressively uniform. The reasoning for these results can be found in the discussion section.

### Wettability (contact angle) measurements

To measure the wettability of the surface, static contact-angle measurements were performed with deionized water (DI) at different locations on the surface of the sample. These values were used to compute the average and standard deviation values of the contact angle (*q* ± 1.0). The addition of SiO_2_ nanocomposites increased the wettability of magnesium (Table S4). The contact angle observed for pure Mg was ∼64° while for composite samples it was ∼58°, 53°, 44° for 0.5%, 1%, 1.5% vol.% SiO_2_ additions, respectively, which are favorable for cell attachment and cell proliferation. The addition of SiO_2_ NPs increased the hydrophilicity of pure Mg. The results highlight the stronger hydrophilicity trait of the samples as more NPs are added.

### Cytotoxicity test

**Fig. 4:**
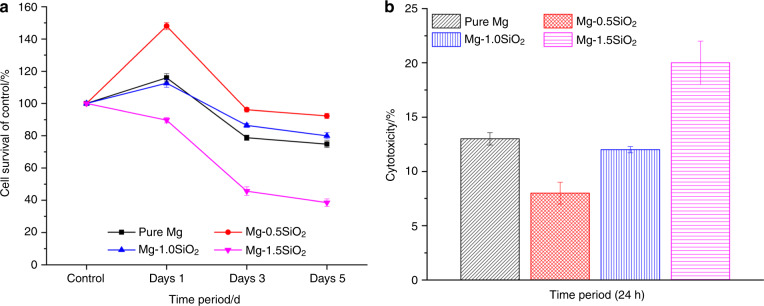
Cytotoxicity in vitro for pure Mg and Mg-SiO_2_ nanocomposites. **a** Remaining cellular activity of and **b** relative cytotoxicity of MC3T3-E1 cells upon exposure to sample discs, as measured by MTS assay and LDH, respectively

Figure 4a shows the results of the MTS assay of Mg-SiO_2_ nanocomposites with MC3T3-E1 pre-osteoblast-like cells. As it is shown, the cells can attach and proliferate at all the concentrations of SiO_2_ but there was an increase in cell proliferation for Mg- (0.5% and 1 vol.%) SiO_2_ nanocomposites. Mg-1.5 vol%. SiO_2_ showed the least proliferation when compared with all the samples on day 3 and day 5. Mg- (0.5% and 1 vol.%) SiO_2_ nanocomposite samples improved and accelerated the cell proliferation compared with the cell growth on Mg-1.5 vol.% SiO_2_ after day 3 and day 5 incubation. Statistical analysis showed no significant difference in cell proliferation on day 1. Nevertheless, after 3 and 5 days, cell proliferation on Mg- (0.5% and 1 vol.%) SiO_2_ nanocomposites were considerably higher in comparison to pure Mg and Mg-1.5 vol.% SiO_2_. The percentage of viable cells was calculated. Mg- (0.5% and 1 vol.%) SiO_2_ nanocomposites showed an increased percentage of cell survival compared with pure magnesium and Mg-1.5 vol.% SiO_2_ (Fig. 4a).

Figure 4b shows an increase in the concentration of SiO_2_ increased the cytotoxicity percentage. Mg-1.5 vol.% SiO_2_ nanocomposite showed more cytotoxicity compared with other groups but it’s within the maximum lactate dehydrogenase (LDH) release concentration of 100%. Mg- (0.5% and 1 vol.%) SiO_2_ nanocomposites showed less cytotoxicity percentage compared with pure Mg. (*p* < 0.05; *n* = 4).

**Fig. 5:**
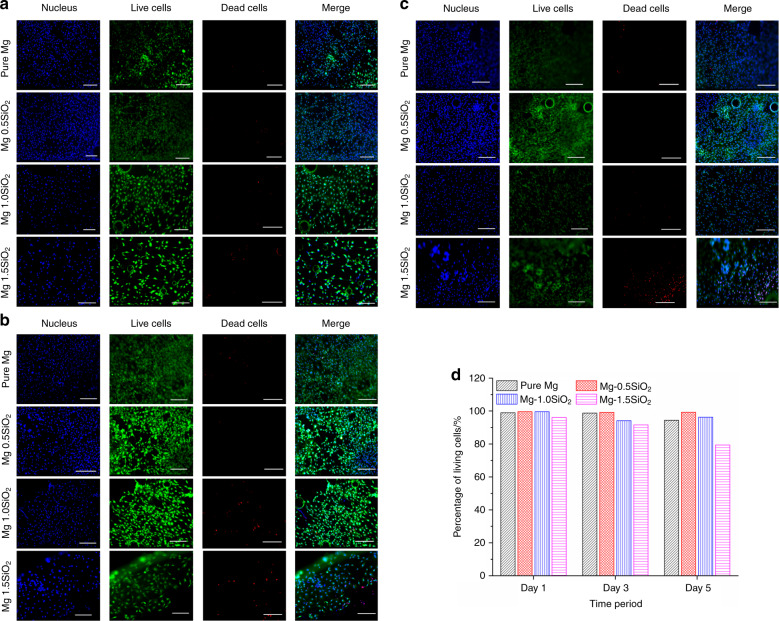
LIVE/DEAD staining of primary MC3T3-E1 cells cultured on samples for **a** Day 1, **b** Day 3, **c** Day 5, respectively, and **d** Percentage of living cells. Viable cells are labeled in green, the nucleus of live cells (blue), dead cells (red) and merge images of live and dead cells. **d** Percentage of living cells. Magnification: ×10 and scale bar are 200 µm

The results showed more live cells (green) and nuclei of live cells (blue) for Mg- (0.5% and 1 vol.%) SiO_2_ compared with Mg-1.5 vol.% SiO_2_ and pure Mg for day 1, 3, and 5 incubation. Cell proliferation and cell density increased from day 1 to 5 for Mg- (0.5% and 1 vol.%) SiO_2_ compared with pure Mg and Mg-1.5 vol.% SiO_2_. Dead cells(red) were more in Mg-1.5 vol.% SiO_2_ nanocomposite on day 3 and day 5 incubation. Less density of dead cells was found for Mg- (0.5% and 1 vol.%) SiO_2_ nanocomposites. The cell distribution was overall better for all the SiO_2_ concentrations as seen in Fig. 5 compared with pure Mg.

**Fig. 6:**
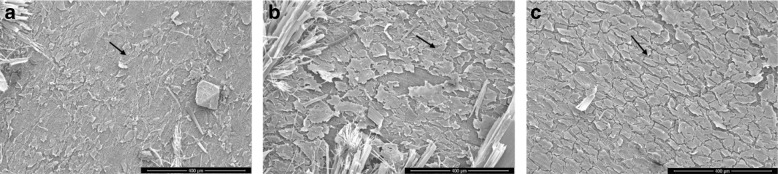
Scanning microscope images of MC3T3-E1 cultured on Mg-SiO_2_ nanocomposite samples for **a** Day 1, **b** Day 3, and **c** Day 7. Arrows show the cell attachment. Scale bars are 400 µm

Typical SEM images of the cells on the samples are shown in Fig. 6, which demonstrates the interaction between the MC3T3-E1 cells and the disc surface as observed on SEM. After day 1 incubation, almost all cells maintained a spindle morphology, and only a few cells presented a slight extracellular membrane bridge for attachment onto the surface of the Mg-SiO_2_ disc. However, numerous cells presented a wide cellular membrane bridge and flattened morphology on the discs on day 3 incubation. These initial cell adhesion results were consistent with the fluorescent image analysis (Fig. 5). Furthermore, after 5 days of incubation, the cells were remarkably elongated and formed an osteoblast-like morphology on all the concentrations of SiO_2_. Cells grew well on the surfaces of all Mg-SiO_2_ composite samples.

### Magnesium vs magnesium/1SiO_2_: stress and magnitude of displacement of customized alloplastic mandibular reconstruction, with the wing design

**Fig. 7:**
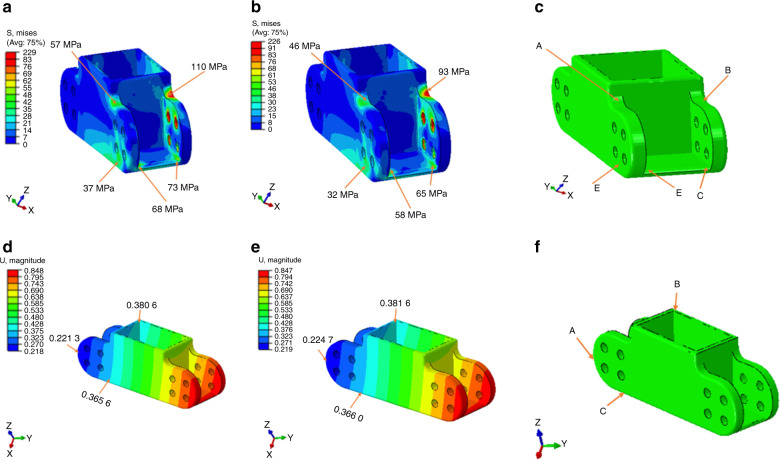
Von Misses stress values (MPa): **a** Pure magnesium, **b** Mg/1 SiO_2_, **c** stress concentration areas. Magnitude of displacement: **d** Pure magnesium, € Mg/1 SiO_2_, **f** Stress concentration areas

Slight downward bending was noted in the mandible initially, however, the mandible arched upwards on both sides under loading conditions (Fig. S5). Even distribution of stresses was observed throughout the endoprosthesis. For the pure magnesium (Group 1), the stress values were in the 37–110 MPa range. The maximum stress values observed at the junction of the body and the stem was 110 MPa. However, the magnesium/1 SiO_2_ (Group 2) exhibited a lower stress value in the 32–93 MPa range. Maximum stress value of 93 MPa was observed at the junction of the wing and the body. Group 1 and group 2 had an average displacement of 0.3–0.6 mm (Fig. 7) (Tables S5, S6).

**Fig. 8:**
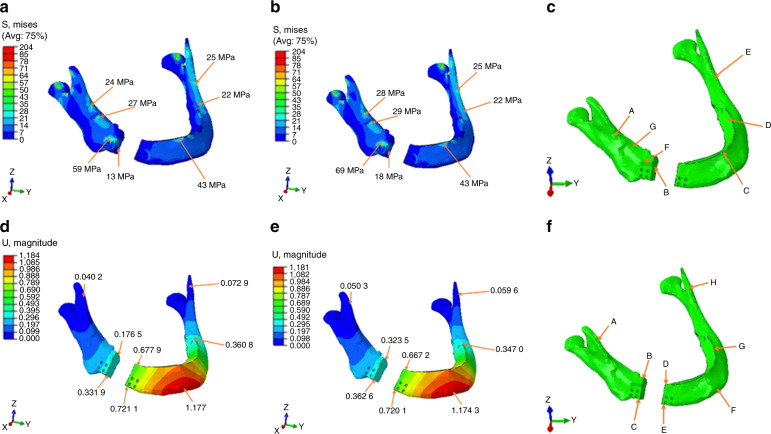
Von Misses stress values of mandible (MPa): **a** Pure magnesium, **b** Mg/1 SiO_2_, **c** stress concentration areas. Magnitude of displacement of mandible: **d** pure magnesium, **e** Mg/1 SiO_2_, **f** stress concentration areas

The stress values on the mandible for the (Group 1) were in the 22–59 MPa range on the defect side and 22–25 MPa range on the unaffected side (left side). Also, the stress values on the mandible for Group 2 were in the 28–69 MPa range on the defect side and in the range of 22–25 MPa range on the unaffected site. The displacement of the mandible near the defect side was <1 mm and the maximum displacement of 1.1 mm was observed near the symphysis region where the load was applied (Fig. 8) (Tables S7, S8). Mg/1SiO_2_ showed lesser stresses concentrated on the endoprosthesis wing compared with the Pure Mg. This implicates the addition of SiO_2_ reduced the stress acting on the endoprosthesis and dissipated the stress evenly throughout the prosthesis. The stresses concentrated on the mandible were almost the same for both the groups.

## Discussion

The objective of this study was to develop Mg-SiO_2_ nanocomposites with improved biomechanical, corrosion, and cytotoxicity properties that can ultimately lead to clinical translation for osteosynthesis and alloplastic replacement that can be resorbed and replaced eventually with bone.

### Characterization of microstructure and mechanical testing

The grain size refinement observed in the Mg-SiO_2_ nanocomposites is primarily attributed to the ability of SiO_2_ NPs to effectively pin the grain boundaries, thereby restricting grain growth. Previous studies on Mg-based nanocomposites reveal that the presence of uniformly dispersed nanosized reinforcements would actively pin the grain boundaries and assist in the nucleation of Mg-matrix grains^10,12,21,22^. The uniform distribution of hollow SiO_2_ NPs in the Mg matrix can be attributed to the isolated clustering effects, if any, owing to the optimized processing and extrusion parameters. In addition, the recrystallization of the as-cast microstructure owing to the high extrusion ratio employed in the hot extrusion method breaks down the clusters/agglomerates and uniformly distributes in the magnesium matrix. This is attributed to the low stacking fault energy of Mg-based alloys^33^. This phenomenon, coupled with the optimized processing parameters like stirring and disintegration, assists in the superior grain refinement^34^. As it can be seen in the SEM micrographs of Mg-SiO_2_ nanocomposites (Fig. S2), the distribution of NPs was found to be near-homogeneous in the Mg matrix.

XRD studies were used to analyze the influence of hollow SiO_2_ NPs on the texture effects of Mg. From the longitudinal X-ray diffractograms (Fig. 1), the intensity corresponding to the pyramidal plane was observed to be increasing for the nanocomposites up to 1 vol.% addition and a relative reduction in basal plane intensity was observed, indicating texture randomization in pure Mg crystal owing to the addition of SiO_2_ NPs. Texture randomization in ceramic-based Mg nanocomposites has been observed previously^20,35^. Table S2 displays values of the maximum XRD intensities and the ratio of the maximum XRD intensity to the respective prismatic, basal, and pyramidal intensities of pure Mg and Mg-SiO_2_ nanocomposites along the longitudinal section. It is observed that the relative basal plane intensity of the nanocomposite decreases with the addition of SiO_2_ NPs (up to 1 vol.%) with minimum I/Imax value being 0.842 exhibited by Mg-1.0 vol.% SiO_2_ nanocomposite. However, further addition of SiO_2_ NPs resulted in a strong basal structure, thereby correlating with the deformability characteristics under compression loading. The randomization in texture owing to the presence and progressive addition of NPs can positively influence the strengthening and plastic deformation characteristics of nanocomposites. The correlation drawn in the current study is discussed briefly in the next paragraphs.

The increase in the strength of the nanocomposite is by the effect of grain refinement by the addition of SiO_2_ (Table S1), helping in the mechanism of Hall-Petch strengthening^21^. The widespread uniform pattern of dispersion of the nanocomposites with the help of Orowan strengthening helps to block the dislocation movement^21^. Texture randomization and deformation twinning can add up strength by preventing crack propagation^10^. Good interfacial bonding enhances the effective load transfer from the matrix to the reinforcement^35^.

From Table S3, the compressive fracture strain values were also found to increase simultaneously along with strength values with the addition of SiO_2_ NPs up to 1 vol.%. This simultaneous enhancement is due to the combined effect of texture changes along with increased work hardening and deformation under compressive load with the addition of SiO_2_ NPs^10,12^. A residual dislocation loop is formed around each SiO_2_ NP activating Orowan strengthening. The Orowan strengthening contribution results in enhanced work hardening, resulting in improved fracture strain values^10,12^. However, with the addition of 1.5 vol.% SiO_2_ NPs, the fracture strain value observed a slight decrease of ~14.6% w.r.t pure Mg. This behavior can be primarily attributed to the presence of few agglomerated sites at higher volume additions of the NPs and their inability to weaken the texture of Mg beyond a threshold point, in this case, ≥1.0 vol.% addition, thereby affecting the plastic deformation capabilities of the material. These agglomerated sites may act as stress concentration sites causing the premature failure of the material. The incorporation of layered fashion of raw material addition during the primary processing and the high extrusion ratio used in this study results in lower agglomerations or clusters. However, the fracture strain value for this composition is still higher than the commercially used biomedical Mg alloys. The key importance of biodegradable materials like Mg-based alloys is that the material should support the load-bearing during the reconstruction/fracture fixation procedure. Hence, high strength and fracture strain properties are essential. Mg-based implants and stents may display a 15–20% reduced fracture strain in a simulated body fluid environment as compared with air^36^. Hence, in terms of biosafety, higher fracture strain properties, as displayed by the Mg-SiO_2_ nanocomposites becomes even more imperative.

### Immersion studies

The primary corrosion type observed in Mg-based materials is pitting corrosion^37^. In the present study, pits of different sizes on the surface of the samples were observed (Fig. 3). However, the presence of SiO_2_ NP decreased the extent of pitting owing to the near-uniform distribution of the NPs in the matrix and reduced grain size^21^. Results also showed a more-uniform passive layer formation for the nanocomposites, which acted as a barrier between the matrix material and the immersive medium thereby delaying the onset of corrosion^10,12^. Among the composite samples, the number of pits observed in Mg-1.5 SiO_2_ were comparatively higher than Mg-0.5 SiO_2_ and Mg-1SiO_2_ composites. The layer formation process appears to be expedited owing to the presence of NP in the immersive medium. Post immersion SEM images are shown in Fig. 3. The Mg(OH)_2_ layer formation in the pure Mg sample is non-uniform and several brucite crystal formations are observed. The brucite crystals are needle-shaped and are made of Mg(OH)_2_. Presence of these needle-shaped structures could be detrimental to the material as it encourages non-uniform corrosion of Mg. Among composites, 0.5 and 1.0 SiO_2_ nanocomposites showed a comparatively more-uniform layer formation when compared with Mg-1.5 SiO_2_ nanocomposite where uniform layer was restricted to only small pockets across the sample. Post layer formation, the Cl^−^ anions react with the Mg^2+^ to form a non-protective MgCl_2_ compound. This compound attacks the hydroxide layer and peels off the layer along the grain boundaries. As observed in Fig. 3b, c, the chloride attacks the grain boundaries as the NPs are present at the grain boundaries peeling off one grain at a time. This process is delayed due to the presence of refined grains developed owing to the NP addition. Among the composite samples, Mg-0.5 SiO_2_ nanocomposite showed minimum corrosion rate while for Mg-1SiO_2_ and Mg-1.5 SiO_2_ nanocomposites, the corrosion rate was slightly higher (Fig. 2). This can be attributed to: (a) the increasing presence of clusters with the increasing amount of SiO_2_ and (b) increase in the amount of cluster associated porosity and dislocation density due to the increasing presence of SiO_2_. To note that the corrosion response observed in the nanocomposites is still better than that of pure Mg.

In addition to the controlled degradation behavior, any potential biomaterial should also encourage in vitro bioactivity to ease the bone resportion process. The ability of the material to enhance the apatite formation on its surface is critical as it dictates the amount of time the host body will take for the bone regrowth^38,39^. EDS analysis of the sample was performed to understand the quantitative presence of the apatite compound formation (Fig. S4). The mapping results of the sample reveal a high amount of Mg, O, and P. This result can indicate the formation of magnesium-based phosphate compounds that assist in the apatite formation. EDS mapping of Mg-1.5 SiO_2_ also shows the compositional difference between the matrix (1), quasi adherent layer (2), and the hydroxide layer (3). The amount of Mg decreases from area 1–3. At the same time, the amount of O and P from the matrix to the hydroxide layer has progressively increased. This behavior highlights the fact that the protective layer present has higher amounts of O and P and is feasible for apatite layer formation.

### Cytocompatibility tests

The functional and esthetic outcomes for orthopedic and mandibular reconstruction are closely related to when selecting a suitable biomaterial. The criteria and goal for a successful reconstruction are to improve facial contours, restore form and function, establish alveolar bone height and width, and establish continuity of the bone. Developing a biomimetic bone substitute that mimics the structural architecture, physical, and chemical properties of the mandibular bone would be the most promising challenge for a clinician. Magnesium-enriched hydroxyapatite biomaterials have been shown to promote bone tissue regeneration, and have excellent biocompatibility and good osteogenic potential when used as a bone substitute for ameloblastoma excision surgeries for mandibular reconstruction^40^. Biodegradable magnesium alloy bone screws are used for fracture fixation of mandibular condyle^41^. Magnesium-based resorbable screws show excellent biocompatibility and biomechanical stability when used for bilateral sagittal split ramus osteotomy^42^. The Mg-SiO_2_ used in this study possesses the ability to mimic the mechanical strength of the mandibular bone and showed excellent biocompatibility.

Biosafety of a potential orthopedic or craniomaxillofacial material is studied by biocompatibility testing^43,44^. The cytotoxicity and cell proliferation studies are the most crucial and widely accepted protocol for a cytocompatibility test owing to its rapidness, sensitivity, and simplicity^45^. In the present study, in vitro cytotoxicity and cell proliferation were done using LDH enzyme release assay and MTS assay experiments indicated a favorable performance of the magnesium nanocomposites containing different compositions of hollow SiO_2_ NPs. The in vitro cytocompatibility and cell viability were determined by direct cell attachment on the samples. We found that the addition of nano hollow SiO_2_ NPs to pure Mg did not alter the cytocompatibility of the composites. With the addition of 0.5 and 1 vol.% SiO_2_, the proliferation of MC3T3-E1 cells showed significant cell proliferation. However, increasing the concentration to 1.5 vol.% showed a decrease in the proliferation rate (Fig. 4). Among the samples tested, cell viability data showed that 0.5 and 1 vol.% SiO_2_ exhibited the lowest cytotoxicity and increased cell viability percentage while the 1.5% SiO_2_ demonstrated a significantly reduced cell viability percentage (Fig. 4a). LDH enzyme release assay results showed that MC3T3-E1 cells cultured in the presence of low and high concentrations of hollow SiO_2_ NPs for 24 h support the cytocompatibility (Fig. 4b). No evident toxic responses were observed in this study. This behavior is akin to the improvement in the corrosion resistance accomplishing higher cell attachment, proliferation, and direct cell viability. Nanosized SiO_2_ exhibits more toxicity than its micron size counterparts as reported in previous investigations^46–48^. An increase in concentration of SiO_2_ NPs possessed a more toxic effect on MC3T3-E1 cells. A comparable response of the cell lines to SiO_2_ suggests a difference in the sensitivity of cells toward the same particles with increased concentrations. These results were consistent with the findings of Lanone et al.^49^ also reported differential sensitivity of human alveolar and macrophage cell lines toward various NPs. The low cell density on pure Mg may be due to the high corrosion rate as well as a higher rate of hydrogen evolution when it is exposed to a physiological environment^50^.

Day 1 and Day 3 incubation showed a change in cell morphology from spindle shape to flattened morphology (Fig. 6). There was a change in the morphology of cells indicating the adhesion process of cells to the surface of the discs. The addition of SiO_2_ as a reinforcement enhances cell addition and promotes change in morphology from day 1 to 5 incubation. SiO_2_ and Mg ions enhance the cell attachment and provide a beneficiary initial stage bone formation and enhance the binding of cell surface receptors and ligand proteins^51,52^. SiO_2_ NPs initiates the cell attachment of MC3T3 and also increases cell proliferation rate as reported previously^53^.

Many factors govern the adhesion of MC3T3-E1 to the implant surface namely surface chemistry, charge or hydrophilicity, and roughness. The literature review suggests that SiO_2_ NPs coatings can enhance the MC3T3 cell attachment resulting in higher cell proliferation for increasing culture periods^53^. Similar observations reported in the present work further emphasize the beneficial role of SiO_2_ NPs in improving the biocompatibility of Mg when reinforced in the form of NPs. Further, enhanced hydrophilic nature of the nanocomposite surface with the presence and progressive addition of SiO_2_ NPs (Table S4) results in the increased affinity to adsorbed water hence promoting the interfacial reaction between the surface and protein, favoring cellular response^54^. Although the cells are adhering to both pure Mg (relatively hydrophobic) and Mg-SiO_2_ nanocomposite surfaces, constant contact is necessary between the cell and the pure Mg substrate for cell division and proliferation contributing to lower cell attachment over nanocomposite samples.

### Finite element analysis for mandibular wing prosthesis

The mandible is subjected to various forces acting on it during mastication and at rest. Four main loads are acting on the mandible, out of which compression/tension and shear forces are linear and the torsion and bending are angular loads^55^. Various methods had been used to study the bodily movement of the mandible in regard to the applied forces, however, the exact one confirmatory method for finding out the displacement and stress acting on the mandible is far impossible^56^. Finite element analysis was used in this study because it is a numerical method of analyzing the stress acting within the prosthesis and modeling of the complex structures could be done easily. A continuity defect created on the right side of the mandible body region was fitted with a wing design endoprosthesis used from our previous studies^57^. The average occlusal maximum bite force of human mandible body with teeth is in the range of 100–4341 N with an average of 750 N^58–62^. The teeth were removed from the mandibular model for easy calibration and modeling. The materials properties of cortical and cancellous bone for mandible and pure Mg and Mg/SiO_2_ was incorporated into the wing design^57^. The occlusal loading of 300 N was selected in accordance with studies showing the average maximum biting forces developed from patients with reconstructed mandible owing to malignancies^63^. The stress distribution acting within the prosthesis and the magnitude of displacement of the mandible were tabulated (Tables S5, S8). Mg/1SiO_2_ showed even stress distribution and the number of stresses acting within the prothesis was less. In comparison, pure Mg showed increased stress concentration within the prosthesis and more amount of stress accumulation at the junction of the body and the wing (Fig. 7). The stresses acting within the mandible were almost the same for both the groups, however, it was slightly more for the Mg/1SiO_2_ group. This is attributed to the fact that more amount of stresses developed within the prosthesis was evenly dissipated to the bone rather than accumulating within the prosthesis. The magnitude of displacement of the prosthesis was <1 mm for both the groups and this could prevent the deleterious stresses within the prosthesis leading to fracture (Fig. 8).

## Conclusions

Motivated by the attractive properties of magnesium in mandibular reconstruction, Mg-SiO_2_ nanocomposites with low-volume fraction addition of hollow silica NPs were developed. The mechanical properties of Mg-SiO_2_ nanocomposites synthesized in the present study are comparable to those of commercially available Mg alloys and mandibular bone with an acceptable corrosion rate. In addition, Mg- (0.5 and 1.0 vol.%) SiO_2_ nanocomposites exhibited no cytotoxicity to MC3T3-E1 cells. Continuous degradation of the nanocomposite implant in vitro could be found, and signs of localized corrosion could also be observed from the SEM analysis. In summary, Mg-SiO_2_ nanocomposites exhibit great potential for use as implant materials in mandibular reconstruction on the condition that SiO_2_ content should be carefully controlled (limited to 1 volume percent).

## Materials and methods

### Material preparation

Magnesium turnings with a purity of >99.9% (trace impurities of Si, Mn, Cu, Al, Fe, Pb, Ni, Sn ≤0.10%) was used as the raw material (ACROS Organics, USA) and the required amount of hollow silica (SiO_2_) NPs in ~10–20 nm size range and purity >99.2 supplied by Sigma Aldrich, Singapore, was used as the reinforcement phase.

The DMD technique was used to synthesize Mg-SiO_2_ nanocomposites^39,64,65^. Pure Mg turnings with weighed amounts of SiO_2_ NPs based on volume fraction required were heated to 750 °C in an argon atmosphere. Uniform dispersion of hollow SiO_2_ NPs in the Mg matrix is ensured by stirring the slurry at 465 r·min^−1^ for 5 min. Thereafter, the molten metal was bottom poured into a metallic mold in the presence of argon gas flowing at 25 liters per minute flow rate to disintegrate the melt steam. The cylindrical preform of 40 mm diameter was cast, homogenization was carried out at 400 °C for 1 h and hot extruded at 350 °C at an extrusion ratio of 20.25:1. Cylindrical rods of 8 mm diameter were obtained and were characterized using ASTM standards.

### Materials characterization

#### Characterization of microstructure and mechanical testing

The microstructure of the samples was studied on the samples post polishing to evaluate the grain size. After polishing, acetic acid was used for light etching of the samples to study the grain size distribution in the Mg matrix. Grain size analysis was performed using the OLYMPUS metallographic optical microscope. NP distribution was studied using the JEOL JSM-6010 scanning electron microscope.

The X-ray diffraction studies of the samples in the as-extruded condition were performed along the extrusion direction. Shimadzu Lab-XRD 6000 diffractometer using Cu K_α_ radiation of wavelength 1.541 8 Å at a scan speed of 2^o^/min was used.

A fully automated servo-hydraulic mechanical testing machine (MTS 810) was used to test the samples for compressive response at a strain rate of 5 × 10^−3^ min^−1^. Aspect ratio (l/d) of 1 was used as per the ASTM test method E9-09. Five samples were tested to ensure reproducibility.

#### Immersion studies

The degradation behavior of pure Mg and Mg-SiO_2_ nanocomposites were conducted by immersion testing in Hank’s balanced salt solution. Hank’s solution used as the medium consisted of 8.0 g·L^−1^ NaCl, 0.4 g·L^−1^ KCl, 0.35 g·L^−1^ NaHCO_3_, 1.0 g·L^−1^ dextrose (C_6_H_12_O_6_), 0.09 g·L^−1^ Na_2_HPO_4_.7H_2_O, 0.06 g·L^−1^ KH_2_PO4, and 0.02 g·L^−1^ C_19_H_14_O_5_S in distilled water. The samples in as-extruded condition for each composition were immersed for 1, 2, 3, 4, and 7 days in a water bath at 37 °C to mimic the temperature conditions of the human body. The ratio between the sample to the solution was adjusted to 20 mL:1 cm^2^. The solution was replaced every 24 h. Post 24 h, weight loss, and pH measurements were done. A solution mixture of 20 g CrO_3_ and 1.9 g AgNO_3_ dissolved in 100 mL of deionized water was used to remove the corrosion products. SEM and EDS were done on the post 7 day corroded samples to know the nature of corrosion products formed.

#### Wettability (contact angle) studies

Contact-angle measurements were done at room temperature using a 10 µl droplet of deionized water addition on the surface of the samples using Drop Shape Analyzer DSA25, Kruss GmbH, Germany. Ten readings per sample were measured.

#### Cytocompatibility

For the direct assay, 5 mm × 2 mm discs were used. Cells were directly seeded on the discs. Cell proliferation was assessed by absorbance values and the percentage of viable cells was calculated from the obtained values. Cell Titer 96^®^ Aqueous Assay System, Promega was used for measuring the cell viability and proliferation. MC3T3-E1 (8000 cells) were seeded directly on the discs in 96 well plate. A standard protocol for the MTS assay was followed according to the manufactures recommendations. Optical density values were measured in 96 well plate reader at 490 nm wavelength. (*P* < 0.05; *n* = 4). The LDH activity was used as an index of cytotoxicity. CytoTox-One Reagent (Promega) was used for LDH assay. The experiments were done according to the manufacture protocol and the fluorescence values were recorded with excitation of 560 nm and an emission wavelength of 590 nm. (*P* < 0.05; *n* = 4). Live-dead cell staining was done using propidium iodide (PI) (Dead cells) stock solution, fluorescein diacetate, and Hoechst (Blue) and examined using an upright fluorescence microscope (Leica DMRB, Leitz). Cell attachment and cytoskeleton morphology were observed using scanning electron microscopy.

#### Modeling and finite element analysis

The study design and the modeling methods were used from our previous studies^57^. The models were re-meshed with 3-matics (V8.0) (Materialise, Leuven, Belgium) to make them more regular. The mesh of the mandible model with the prosthesis was composed of 447 669 nodes and 315 647 elements (Table S9 and Fig. S6). From our previous study, we showed that the performance of the whole reconstruction was not just dependent on the design. We used the information derived from the mechanical testing to see if this material could withstand the masticatory forces. So, an average masticatory load of 300 N was applied to the incisor region using finite element analysis software (Abaqus)^57^.

## Supplementary information

Additional Supplementary Material

Supplemental Material File #1

Supplemental Material File #2
