# Supplementary material for: Hollow silica reinforced magnesium nanocomposites with enhanced mechanical and biological properties with computational modeling analysis for mandibular reconstruction
Source: Int J Oral Sci. 2020 Nov 17;12:31. doi: 10.1038/s41368-020-00098-x (PMC7673133; doi:10.1038/s41368-020-00098-x)
Supplement: Supplementary file 1 — Additional Supplementary Material [file 41368_2020_98_MOESM1_ESM.docx]

**SUPPLEMENTARY TABLES AND FIGURES**

Table S1. Results of grain size measurements for developed materials.

| **Materials** | Pure Mg | Mg-0.5SiO_2_ | Mg-1.0SiO_2_ | Mg-1.5SiO_2_ |
| --- | --- | --- | --- | --- |
| **Grain size (µm)** | 36 ± 4 | 25 ± 4.5(↓30.5%) | 21 ± 2.5(↓41.7%) | 16 ± 2.8(↓55.6%) |

Table S2. X-Ray Diffractogram results of as-extruded Mg-SiO_2_ nanocomposites.

| **Material** | **Plane** | **I/Imax** |
| --- | --- | --- |
| Pure Mg | 10-10 Prism  0002 Basal  10-11 Pyramidal | 0.154  1.000  0.794 |
| Mg-0.5 SiO_2_ | 10-10 Prism  0002 Basal  10-11 Pyramidal | 0.154  1.000  0.927 |
| Mg-1.0 SiO_2_ | 10-10 Prism  0002 Basal  10-11 Pyramidal | 0.233  0.842  1.000 |
| Mg-1.5 SiO_2_ | 10-10 Prism  0002 Basal  10-11 Pyramidal | 0.110  1.000  0.456 |

Table S3. Contact angle measurements of the developed materials.

| **Material** | Pure Mg | Mg-0.5SiO_2_ | Mg-1.0SiO_2_ | Mg-1.5SiO_2_ |
| --- | --- | --- | --- | --- |
| **Contact angle (Degrees)** | 62±1 | 58±1 | 53±1 | 44±1 |

Table S4 : Magnitude of Displacement of Wing prosthesis

|  | **DIRECTION** | **LOCATIONS** | | |
| --- | --- | --- | --- | --- |
|  |  | **A** | **B** | **C** |
| **Mg / 1 SiO_2_** | x | 0.0063 | 0.0663 | 0.0663 |
|  | y | 0.1118 | 0.1302 | 0.1302 |
|  | z | 0.1928 | 0.3525 | 0.3525 |
|  | mag | 0.2247 | 0.3816 | 0.3816 |
|  |  |  |  |  |
| **Pure Mg** | x | 0.0058 | 0.0674 | 0.0586 |
|  | y | 0.1090 | 0.1288 | 0.1135 |
|  | z | 0.1952 | 0.3517 | 0.3426 |
|  | mag | 0.2213 | 0.03806 | 0.3656 |

Table S5 : Von Misses Stress values on the mandible

|  | **LOCATIONS** | | | | | | |
| --- | --- | --- | --- | --- | --- | --- | --- |
|  | **A** | **B** | **C** | **D** | **E** | **F** | **G** |
|  | **MPa** | **MPa** | **MPa** | **MPa** | **MPa** | **MPa** | **MPa** |
| **Mg / 1 SiO_2_** | 28 | 18 | 43 | 22 | 25 | 69 | 29 |
| **Pure Mg** | 24 | 13 | 43 | 22 | 25 | 59 | 27 |

Table S6 : Magnitude of Displacement of mandible

|  | **DIRECTION** | **LOCATIONS** | | | | | | | |
| --- | --- | --- | --- | --- | --- | --- | --- | --- | --- |
|  |  | **A** | **B** | **C** | **D** | **E** | **F** | **G** | **H** |
| **Mg / 1 SiO_2_** | **x** | 0.0174 | 0.0426 | 0.0748 | 0.1116 | 0.1972 | 0.2631 | 0.1244 | 0.0105 |
|  | **y** | 0.0061 | 0.0857 | 0.1937 | 0.0123 | 0.1705 | 0.0528 | 0.1054 | 0.0424 |
|  | **z** | 0.0468 | 0.3080 | 0.2972 | 0.6576 | 0.6712 | 1.1435 | 0.3063 | 0.0422 |
|  | **mag** | 0.0503 | 0.3235 | 0.3626 | 0.6672 | 0.7201 | 1.1743 | 0.3470 | 0.0596 |
|  |  |  |  |  |  |  |  |  |  |
| **Pure Mg** | **x** | 0.0105 | 0.0301 | 0.0522 | 0.1046 | 0.1988 | 0.2632 | 0.1284 | 0.0096 |
|  | **y** | 0.0155 | 0.0665 | 0.1054 | 0.0109 | 0.1694 | 0.0512 | 0.1044 | 0.0524 |
|  | **z** | 0.0355 | 0.1607 | 0.3104 | 0.6697 | 0.6721 | 1.1461 | 0.3205 | 0.0498 |
|  | **mag** | 0.0402 | 0.1765 | 0.3319 | 0.6779 | 0.7211 | 1.777 | 0.3608 | 0.0729 |

Table S7 : Mesh models Nodes and Elements

| **MODEL** | **NODES** | **ELEMENTS** |
| --- | --- | --- |
| WING DESIGN | 151341 | 89208 |
| MANDIBULAR BONE | 381399 | 270944 |
| WING DESIGN WITH MANDIBULAR BONE MODEL | 447669 | 315647 |

**SUPPLEMENTARY FIGURES**

Figure S1. Microscopic images showing grain characteristics of (a) Pure Mg, (b) Mg-0.5SiO_2_, (c) Mg-1 SiO_2_ and (d) Mg-1.5 SiO_2_ nanocomposites. Scale Bar :100 μm

Figure S2. SEM micrographs of (a) SiO_2_ distribution and (b) EDS mapping in Mg-1.0 SiO_2_ nanocomposite.





Figure S3. Stress-Strain graph of Mg and Mg-SiO_2_ nanocomposites.

Figure S4. EDS mapping of the corroded surface for Mg-1.5SiO_2_ nanocomposite.

(A) (B)

Figure S5 : (A) 300-N vertical loads applied in the symphysis region ,(B) Mesh Model of the mandible


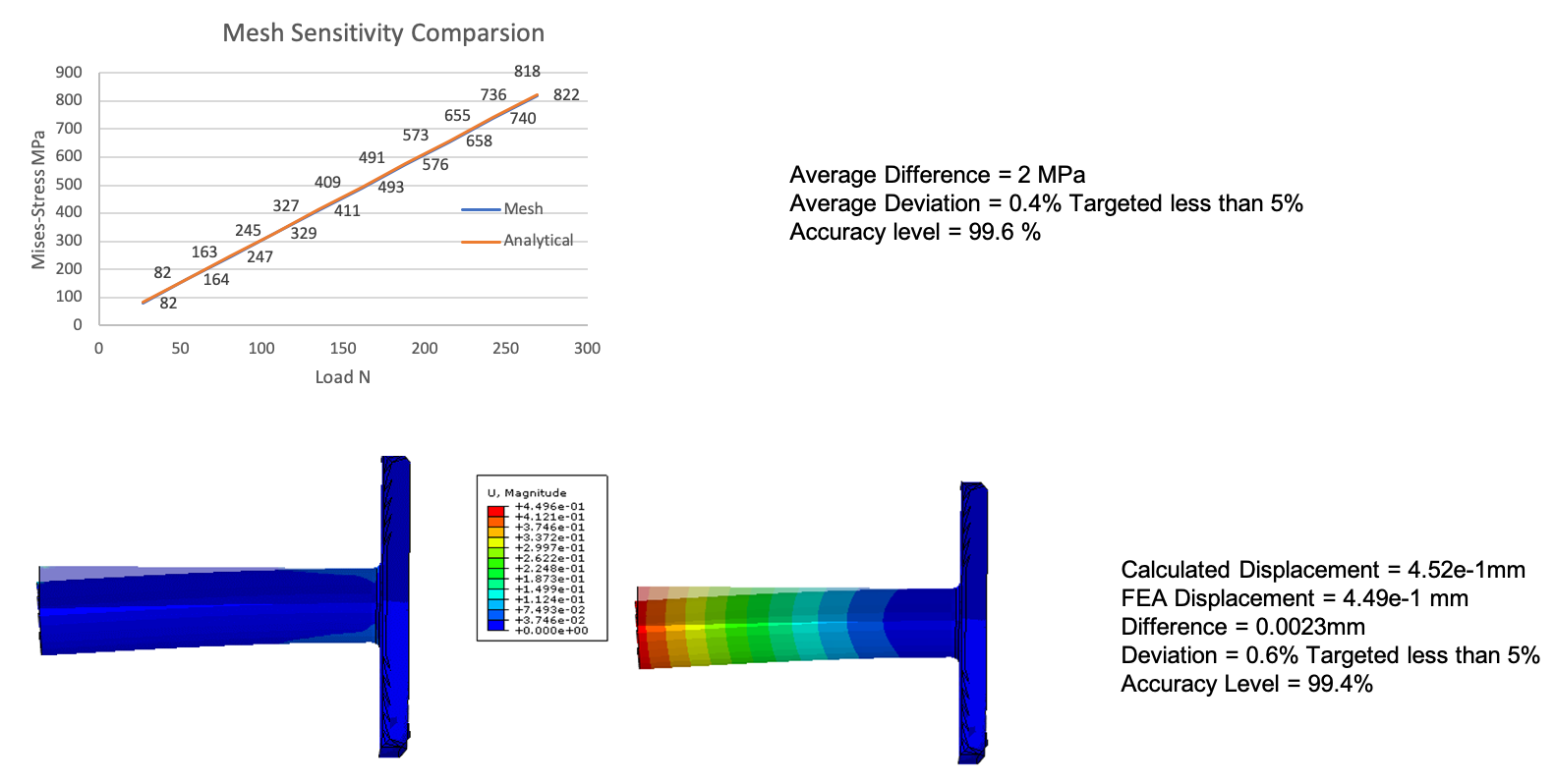


Figure S6 : Mesh Sensitivity Analysis.
