# Supplementary material for: Hollow silica reinforced magnesium nanocomposites with enhanced mechanical and biological properties with computational modeling analysis for mandibular reconstruction
Source: Int J Oral Sci. 2020 Nov 17;12:31. doi: 10.1038/s41368-020-00098-x (PMC7673133; doi:10.1038/s41368-020-00098-x)
Supplement: Supplementary file 2 — Supplemental Material File #1 [file 41368_2020_98_MOESM2_ESM.docx]

**SUPPLEMENTARY TABLES AND FIGURES**

Table S1: Results of grain size measurements for developed materials.

| **Materials** | Pure Mg | Mg-0.5SiO_2_ | Mg-1.0SiO_2_ | Mg-1.5SiO_2_ |
| --- | --- | --- | --- | --- |
| **Grain size (µm)** | 36 ± 4 | 25 ± 4.5(↓30.5%) | 21 ± 2.5(↓41.7%) | 16 ± 2.8(↓55.6%) |

Table S2: X-Ray Diffractogram results of as-extruded Mg-SiO_2_ nanocomposites.

| **Material** | **Plane** | **I/Imax** |
| --- | --- | --- |
| Pure Mg | 10-10 Prism  0002 Basal  10-11 Pyramidal | 0.154  1.000  0.794 |
| Mg-0.5 SiO_2_ | 10-10 Prism  0002 Basal  10-11 Pyramidal | 0.154  1.000  0.927 |
| Mg-1.0 SiO_2_ | 10-10 Prism  0002 Basal  10-11 Pyramidal | 0.233  0.842  1.000 |
| Mg-1.5 SiO_2_ | 10-10 Prism  0002 Basal  10-11 Pyramidal | 0.110  1.000  0.456 |

Table S3: Comparison of compressive properties among nanocomposites in the

present study, commercially available Mg alloys and alternative materials developed

for craniomaxillofacial osteosynthesis applications (adopted from^8^).

| **Materials** | **0.2 CYS (MPa)** | **UCS (MPa)** | **Fracture**  **strain (%)** |
| --- | --- | --- | --- |
| Pure Mg | 65 ± 1 | 297 ± 4 | 21.2 ± 0.4 |
| Mg-0.5 SiO_2_ | 104 ± 2  (↑60%) | 313±3  (↑5.4%) | 21.8 ± 0.3  (↑2.8%) |
| Mg-1.0 SiO_2_ | 122 ± 2  (↑87.7%) | 324 ± 4  (↑9.1%) | 23.8 ± 0.2  (↑12.2%) |
| Mg-1.5SiO_2_ | 128 ± 3  (↑97%) | 378 ± 6  (↑27.2%) | 18.1 ± 0.6  (↓14.6%) |
| Natural bone | 130-180 | - | - |
| Cortical bone | - | 131-224 | 2-12 |
| AM50 | 110 | 312 | 11.5 |
| AZ91D | 130 | 300 | 12.4 |
| WE43 | 183 | 305 | 11.3 |
| ZK60 | 159 | 472 | 12.4 |
| Ti-6Al-4V alloy | 970 | - | - |
| 316L Stainless Steel | 170-310 | - | - |

Table S4: Contact angle measurements of the developed materials.

| **Material** | Pure Mg | Mg-0.5SiO_2_ | Mg-1.0SiO_2_ | Mg-1.5SiO_2_ |
| --- | --- | --- | --- | --- |
| **Contact angle (Degrees)** | 62±1 | 58±1 | 53±1 | 44±1 |

Table S5: Von Misses Stress values on the Prosthesis

| **Materials** | **LOCATIONS** | | | | |
| --- | --- | --- | --- | --- | --- |
|  | **A** | **B** | **C** | D | E |
|  | **MPa** | **MPa** | **MPa** | **MPa** | **MPa** |
| **Mg-1SiO_2_** | 46 | 93 | 65 | 58 | 32 |
| **Pure Mg** | 57 | 110 | 73 | 68 | 37 |

Table S6: Magnitude of Displacement of Wing prosthesis

|  | **DIRECTION** | **LOCATIONS** | | |
| --- | --- | --- | --- | --- |
|  |  | **A** | **B** | **C** |
| **Mg / 1 SiO_2_** | x | 0.0063 | 0.0663 | 0.0663 |
|  | y | 0.1118 | 0.1302 | 0.1302 |
|  | z | 0.1928 | 0.3525 | 0.3525 |
|  | mag | 0.2247 | 0.3816 | 0.3816 |
|  |  |  |  |  |
| **Pure Mg** | x | 0.0058 | 0.0674 | 0.0586 |
|  | y | 0.1090 | 0.1288 | 0.1135 |
|  | z | 0.1952 | 0.3517 | 0.3426 |
|  | mag | 0.2213 | 0.03806 | 0.3656 |

Table S7: Von Misses Stress values on the mandible

|  | **LOCATIONS** | | | | | | |
| --- | --- | --- | --- | --- | --- | --- | --- |
|  | **A** | **B** | **C** | **D** | **E** | **F** | **G** |
|  | **MPa** | **MPa** | **MPa** | **MPa** | **MPa** | **MPa** | **MPa** |
| **Mg / 1 SiO_2_** | 28 | 18 | 43 | 22 | 25 | 69 | 29 |
| **Pure Mg** | 24 | 13 | 43 | 22 | 25 | 59 | 27 |

Table S8: Magnitude of Displacement of mandible

|  | **DIRECTION** | **LOCATIONS** | | | | | | | |
| --- | --- | --- | --- | --- | --- | --- | --- | --- | --- |
|  |  | **A** | **B** | **C** | **D** | **E** | **F** | **G** | **H** |
| **Mg / 1 SiO_2_** | **x** | 0.0174 | 0.0426 | 0.0748 | 0.1116 | 0.1972 | 0.2631 | 0.1244 | 0.0105 |
|  | **y** | 0.0061 | 0.0857 | 0.1937 | 0.0123 | 0.1705 | 0.0528 | 0.1054 | 0.0424 |
|  | **z** | 0.0468 | 0.3080 | 0.2972 | 0.6576 | 0.6712 | 1.1435 | 0.3063 | 0.0422 |
|  | **mag** | 0.0503 | 0.3235 | 0.3626 | 0.6672 | 0.7201 | 1.1743 | 0.3470 | 0.0596 |
|  |  |  |  |  |  |  |  |  |  |
| **Pure Mg** | **x** | 0.0105 | 0.0301 | 0.0522 | 0.1046 | 0.1988 | 0.2632 | 0.1284 | 0.0096 |
|  | **y** | 0.0155 | 0.0665 | 0.1054 | 0.0109 | 0.1694 | 0.0512 | 0.1044 | 0.0524 |
|  | **z** | 0.0355 | 0.1607 | 0.3104 | 0.6697 | 0.6721 | 1.1461 | 0.3205 | 0.0498 |
|  | **mag** | 0.0402 | 0.1765 | 0.3319 | 0.6779 | 0.7211 | 1.777 | 0.3608 | 0.0729 |

Table S9 : Mesh models Nodes and Elements

| **MODEL** | **NODES** | **ELEMENTS** |
| --- | --- | --- |
| WING DESIGN | 151341 | 89208 |
| MANDIBULAR BONE | 381399 | 270944 |
| WING DESIGN WITH MANDIBULAR BONE MODEL | 447669 | 315647 |
