# Supplementary material for: Hollow silica reinforced magnesium nanocomposites with enhanced mechanical and biological properties with computational modeling analysis for mandibular reconstruction
Source: Int J Oral Sci. 2020 Nov 17;12:31. doi: 10.1038/s41368-020-00098-x (PMC7673133; doi:10.1038/s41368-020-00098-x)
Supplement: Supplementary file 3 — Supplemental Material File #2 [file 41368_2020_98_MOESM3_ESM.docx]

Table S3: Comparison of compressive properties among nanocomposites in the

present study, commercially available Mg alloys and alternative materials developed

for craniomaxillofacial osteosynthesis applications (adopted from^8^).

| **Materials** | **0.2 CYS (MPa)** | **UCS (MPa)** | **Fracture**  **strain (%)** |
| --- | --- | --- | --- |
| Pure Mg | 65 ± 1 | 297 ± 4 | 21.2 ± 0.4 |
| Mg-0.5 SiO_2_ | 104 ± 2  (↑60%) | 313±3  (↑5.4%) | 21.8 ± 0.3  (↑2.8%) |
| Mg-1.0 SiO_2_ | 122 ± 2  (↑87.7%) | 324 ± 4  (↑9.1%) | 23.8 ± 0.2  (↑12.2%) |
| Mg-1.5SiO_2_ | 128 ± 3  (↑97%) | 378 ± 6  (↑27.2%) | 18.1 ± 0.6  (↓14.6%) |
| Natural bone | 130-180 | - | - |
| Cortical bone | - | 131-224 | 2-12 |
| AM50 | 110 | 312 | 11.5 |
| AZ91D | 130 | 300 | 12.4 |
| WE43 | 183 | 305 | 11.3 |
| ZK60 | 159 | 472 | 12.4 |
| Ti-6Al-4V alloy | 970 | - | - |
| 316L Stainless Steel | 170-310 | - | - |

Table S5: Von Misses Stress values on the Prosthesis

| **Materials** | **LOCATIONS** | | | | |
| --- | --- | --- | --- | --- | --- |
|  | **A** | **B** | **C** | D | E |
|  | **MPa** | **MPa** | **MPa** | **MPa** | **MPa** |
| **Mg-1SiO_2_** | 46 | 93 | 65 | 58 | 32 |
| **Pure Mg** | 57 | 110 | 73 | 68 | 37 |
